# Predictors of Early Introduction of Core and Discretionary Foods in Australian Infants—Results from HSHK Birth Cohort Study

**DOI:** 10.3390/nu12010258

**Published:** 2020-01-19

**Authors:** Narendar Manohar, Andrew Hayen, Sameer Bhole, Amit Arora

**Affiliations:** 1 School of Health Sciences, Western Sydney University, Campbelltown Campus, Locked Bag 1797, Penrith, NSW 2571, Australia; a.arora@westernsydney.edu.au; 2 Australian Centre for Public and Population Health Research, Faculty of Health, University of Technology Sydney, Ultimo, NSW 2007, Australia; andrew.hayen@uts.edu.au; 3 Oral Health Services, Sydney Local Health District and Sydney Dental Hospital, Surry Hills, NSW 2010, Australia; sameer.bhole@health.nsw.gov.au; 4 Sydney Dental School, Faculty of Medicine and Health, The University of Sydney, Surry Hills, NSW 2010, Australia; 5 Oral Health Alliance, Oral Health Centre, University of Queensland, Brisbane, QLD 4072, Australia; 6 Metro North Oral Health Services, Stafford, QLD 4053, Australia; 7 Translational Health Research Institute, Western Sydney University, Campbelltown Campus, Locked Bag 1797, Penrith, NSW 2571, Australia; 8 Discipline of Child and Adolescent Health, Sydney Medical School, Faculty of Medicine and Health, University of Sydney Faculty of Medicine and Health, Westmead, NSW 2050, Australia

**Keywords:** complementary foods, core foods, discretionary foods, determinants, infants, nutrition

## Abstract

Early introduction of complementary foods can have a detrimental impact on children’s long-term health. This study examined the timing and determinants of early introduction of core and discretionary foods among infants in Sydney, Australia. Mothers (*n* = 1035) from an ongoing population-based birth cohort study were interviewed at 8, 17, 34 and 52 weeks postpartum. The outcome was ‘age at which particular core and discretionary food items were first introduced’. Multivariable logistic regression models were used to investigate family and infant-related determinants of early introduction of core (<17 weeks of age) and discretionary foods (<52 weeks of age). Of the 934 mother-infant dyads interviewed, 12% (*n* = 113) of infants were introduced core foods before 17 weeks of age (median: 22). Mothers working part-time (adjusted odds ratio (OR): 3.42, 95% confidence interval (CI): 1.54–7.62) and those exclusively formula-feeding their babies at four-weeks postpartum (adjusted OR 3.26, 95% CI: 1.99–5.33) were most likely to introduce core foods early. Ninety-five percent (*n* = 858) of infants were introduced discretionary foods before 52 weeks of age (median: 28). Low socio-economic status was significantly associated with early introduction of discretionary foods (adjusted OR: 3.72, 95% CI: 1.17–11.78). Compliance with infant feeding guidelines related to core foods was better; however, discretionary foods were introduced early in most infants.

## 1. Introduction

According to the World Health Organization (WHO), infants should be exclusively breastfed until the age of six months, followed by the introduction of nutritious foods at six months of age to complement on-going breastfeeding [1]. The European Society of Paediatric Gastroenterology Hepatology and Nutrition (ESPGHAN) guidelines [2] state that complementary foods should be introduced “no earlier than 17 weeks and no later than 26 weeks”. In Australia, the recommendations related to timing of introducing complementary foods and the types of foods to be introduced are diverse and to some extent confusing [3]. The 2013 Australian National Health and Medical Research Council (NHMRC) recommendations state that complementary foods should be introduced at around six months of age and nutritious foods can be introduced in any order provided iron-rich foods are the first foods to be introduced to the baby [4,5]. In 2016, an Infant Feeding Summit was held in Australia, which recommended that “When an infant is ready, at around six months, but not before four months, start to introduce a variety of complementary foods (such as iron-rich foods, cooked egg, dairy, wheat products), starting with iron-rich foods, while continuing breastfeeding” [3].

The transition from exclusive breastfeeding or formula-feeding to complementary foods is essential for children’s growth and development as well as their long-term health status [6,7]. Early introduction of complementary foods may lead to nutritional or energy imbalance due to replacement of milk in the diet, potentially increasing the risk of gastrointestinal diseases, and food allergies [8]. It can also lead to chronic diseases such as obesity [9] and early childhood caries (ECC) [10] that are highly prevalent amongst young Australian children [11,12]. Moreover, feeding behaviour in the first year of life sets the stage for a child’s nutritional preferences and habits in later life [13].

The Australian Guide to Healthy Eating (AGHE) [14] classifies foods and drinks (referred to as foods) into two main groups: (1) ‘core foods’, which contain all essential nutrients that a body requires for optimal growth and development and (2) ‘discretionary foods’, which should be avoided in the first year of life [14,15,16,17]. Core foods comprise five major food groups along with water i.e., cereals, fruit, vegetables, meat and alternatives, and milk and alternatives [14,15,16,17] whereas discretionary foods comprise nutrition-poor foods having high levels of saturated fat, added sugar and/or added salt [17]. This categorisation serves as a simple tool to define ‘healthy’ and ‘unhealthy’ foods [18].

In Australia, despite existing guidelines on early infant feeding, recent national and New South Wales (NSW) state reports suggest an overall poor adherence rates amongst Australian families [10,19,20]. In particular, recent statistics from the South Western Sydney (SWS) region of NSW have exhibited a poor compliance with infant feeding recommendations [21,22]. The SWS is a metropolitan area located within the Greater Western Sydney region with an estimated population of 966, 450, which accounts for about 12.5% of the New South Wales (NSW) population [23]. Its resident population is considerably diverse in terms of socioeconomic background and ethnicity [21,23]. There is limited longitudinal data related to the complementary feeding practices’ specifically for the populations residing in SWS region of NSW [24,25,26]. Hence, the aims of this study are:

(a) to assess the timing of introduction of core and discretionary foods in infants residing in SWS region; and
(b) to ascertain the family and infant-related predictors of early introduction of core and discretionary foods in this population group.

The resulting data will indicate the level of compliance with the existing Australian and international infant feeding recommendations. Additionally, it will assist in developing interventions to promote optimal infant feeding practices with respect to the timing of introduction of complementary foods (both core and discretionary) in similar populations across Australia.

## 2. Methods

### 2.1. Sample

This study analyses data from the Health Smiles Healthy Kids (HSHK) birth-cohort study based in SWS, which commenced in late 2009 [25]. The details of the cohort study are described elsewhere [25,27]. In summary, mothers of new-born infants having no known medical condition admitted to public hospitals within the jurisdiction of former Sydney South West Area Health Service (now classified as the Sydney and South Western Sydney Local Health Districts), between October 2009 and February 2010, were contacted to participate in the HSHK study.

Public hospitals were chosen with an intent to recruit under-represented socio-economically disadvantaged population groups and also because the entire Sydney region cannot be covered geographically [28]. Child and Family Health Nurses (CFHNs) during their first post-natal home visit (four to six weeks postpartum) recruited the mother-infant dyads [27,29]. The CFHNs described the study to the mothers and obtained their written informed consent. Interpreter services were arranged for non-English speaking participants and, furthermore, written materials in the respective languages of the major ethnic groups living in SWS region were also provided.

### 2.2. Data Collection

Initially, a baseline telephone interview (at 8 weeks postpartum) was conducted to gather demographic and infant-feeding information after which follow-up telephone interviews were conducted at 17, 34 and 52 weeks postpartum. The study questionnaire was adapted from the Perth Infant Feeding Studies (PIFS I and II) [28,30,31], the Iowa Fluoride study [32], and NSW Child Health Questionnaire [20], to determine if the key core and discretionary foods have been introduced in infants and identify the factors associated with early introduction of these foods. At every interview, information on infant feeding practices including breastfeeding, use of infant formula, and the introduction of key core and discretionary foods, was collected. These two food groups were categorised according to the 2013 AGHE and the 2013 Australian Dietary Guidelines Educator Guide [14,17,33].

Core foods comprised of five food groups in addition to water (tap and bottled): grain (cereal) foods (e.g., cereals, bread, rice, pasta, crackers), fruit (fresh, frozen or tinned), vegetables (fresh, frozen or canned), lean meat (e.g., beef, lamb, pork), poultry, fish (fresh, frozen, or tinned), and eggs, dairy products (e.g., milk, cheese, plain yoghurt).

The discretionary foods were categorised into two main groups: foods high in saturated fats such as biscuits, cakes, puddings, potato chips and savoury snacks. foods and drinks with added sugars such as confectionary, sugar-sweetened beverages (SSBs), flavoured mineral water, sports drinks, sweetened yogurt, ice cream, and syrups or spreads. Moreover, it is recommended that fruit juices should be avoided also [34] because of their high total sugar and energy value [34].

### 2.3. Outcome Measure

Timing of introduction of core and discretionary foods: at every interview phase, the mothers were asked the question “How often was your baby fed the specifically listed foods in the last seven days, and the age in weeks at which they first tried them”. Early introduction of core foods was considered to be before 17 weeks of age, considering the recommendations from the 2016 Australian Infant Feeding Summit [3]. Early introduction of discretionary foods was considered to be before 52 weeks of age [10,14,15,16,17]. These were the two primary outcomes for this study.

The earliest age (in weeks) that the infant had first tried each food type was recorded. The responses were primarily taken from the 17-weeks interview. If the child was not introduced to any food by this period, then the responses were taken from the follow-up interviews at 34 weeks and 52 weeks. This possibly ensured that responses truly reflected the actual time of introduction of core and discretionary foods respectively.

### 2.4. Potential Predictors of Timing

A series of family and infant characteristics were recorded. Family characteristics included maternal age (in years), marital status, mother’s level of education, mother’s employment status (including type of employment) at 17 and 52 weeks postpartum, mother’s country of birth, partner’s country of birth, parity, maternal smoking and alcohol intake during pregnancy, and socioeconomic status (SES). In regard to SES, mothers were enquired about their residential postcode, and their SES was classified based on the Census Index of Relative Socioeconomic Advantage and Disadvantage (IRSAD) [35]. Infant factors included baby’s gender, gestational age, birth weight, delivery method, and feeding method at 4-weeks postpartum.

### 2.5. Statistical Analyses

Stata Statistical Software: Version 15 (StataCorp, College Station, TX, USA) [36] was used to perform the statistical analyses. Descriptive analyses were performed to describe the proportion of infants introduced to core and discretionary foods.

First, univariable logistic regression analyses were performed to examine the factors associated with early introduction of core foods (<17 weeks) and discretionary foods (<52 weeks). Variables were included in the multivariable logistic regression model if their univariable analyses *p*-value was ≤0.25. The multivariable logistic regression analyses were performed to determine variables that were independently associated with the two primary outcome measures using the backward stepwise elimination approach. Only variables that were statistically associated (*p* < 0.05) with the two outcome measures remained in the final model. Dropping of non-significant variables that could potentially affect the model fitness was avoided by assessing the fitness of model at every step of the analyses. All variables in the final model were variables from which, when excluded, the change in log likelihood ratio compared with the corresponding chi-squared (*X*^2^) test statistic on the relevant degrees of freedom was significant.

### 2.6. Ethics Approval and Consent to Participate

Former Sydney South West Area Health Service—RPAH Zone (ID number X08-0115), Liverpool Hospital, University of Sydney, and Western Sydney University provided the ethics approvals for this study. All participants agreed to participate in this study by signing a written consent form.

## 3. Results

Initially, 1500 mothers were contacted to participate in the HSHK study, of whom 1035 provided a written consent (69% response rate). Mothers who declined to participate (*n* = 465) were asked about their socio-demographic details and infant feeding method to determine the representativeness of the sample. Maternal age (*X*^2^ = 4.75, *p* = 0.153), educational level (*X*^2^ = 6.65, *p* = 0.328) and infant feeding method (*X*^2^ = 2.46, *p* = 0.813) were not significantly different between participating and non-participating mothers. Of the 1035 mother-infant dyads recruited, 101 mothers either opted out from the study or could not be contacted via telephone (7 attempts made) prior to the baseline interview. Hence, 934 completed the interviews at 17 and 34 weeks (response rate 62.2%) while the total sample dropped to 900 by the 52-weeks interview (response rate 60%). There were no differences in the age, education level and infant feeding method of those who completed the 52-weeks interview and those who withdrew from the study (data not reported). Family and infant characteristics of the study population are explained in Table 1.

**Figure 1:**
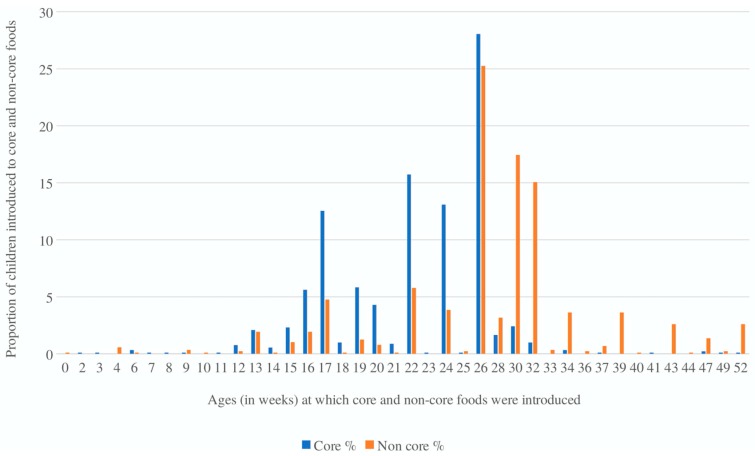
The distribution of age at which core and discretionary foods were first introduced.

In total, 12% (*n* = 112) infants had received core foods before 17 weeks of age. The median age for the introduction of core foods was 22 weeks (interquartile 1 (Q1): 15.5 and interquartile 3 (Q3): 27) with the peak time of introduction at 26 weeks (Figure 1). In relation to discretionary foods, 95.3% (*n* = 858) infants had received discretionary foods before 52 weeks of age. The median age for introduction of discretionary foods was 28 weeks (Q1: 16.5 and Q3: 41) with the peak time of introduction at 26 weeks (Figure 1).

### 3.1. Predictors of Early Introduction of Core Foods

In the univariable model, a variety of family and infant factors were associated with early introduction of core foods (<17 weeks of age) (Table 2). Young (odds ratio (OR) = 0.90, 95% confidence interval (CI): 0.87–0.94; *p* < 0.001)) and single women (OR = 2.97, 95% CI: 1.73–5.09; *p* < 0.001) were more likely to introduce core foods to their infants early. Mothers who were university-educated were less likely to introduce core foods early (OR = 0.34, 95% CI: 0.19–0.59); *p* < 0.001. Women whose partners migrated to Australia from other English-speaking countries were less likely to introduce core foods early (OR = 0.20, 95% CI: 0.08–0.53; *p* = 0.001). Mothers residing in areas considered to be moderately disadvantaged had the highest odds of introducing core foods early (OR = 3.89, 95% CI: 1.45–10.46, *p* = 0.007). Mothers who smoked cigarettes during pregnancy were more likely to introduce core foods to their babies early (OR = 3.57, 95% CI: 0.95–5.70; *p* < 0.001). Regarding infant factors, women who were formula-feeding their babies at four-weeks postpartum were most likely to introduce core foods early (OR = 3.96, 95% CI: 2.52–6.24; *p* < 0.001).

Table 2 presents the adjusted odds ratios (adjOR) for factors that independently predict early introduction of core foods (<17 weeks). The odds of introducing core foods before 17 weeks decreased with increasing maternal age i.e., with every one-year increase in maternal age there was 8% lower likelihood of introducing core foods early (adjOR = 0.92, 95% CI: 0.88–0.96; *p* < 0.001). Mothers who had attained university education (adjOR = 0.53, 95% CI: 0.28–0.99; *p* = 0.04) had lower odds of introducing core foods early compared to those who did not complete school. Women who were working part-time by the 17-week interview had three times the odds of introducing core foods early (adjOR = 3.42, 95% CI: 1.54–7.62; *p* = 0.003). Compared to women born in Australia, migrant women from non-English countries had lower odds of introducing core foods early (adjOR = 0.53, 95% CI: 0.32–0.86; *p* = 0.01) whereas those who migrated from other English-speaking countries had higher odds of introducing core foods early (adjOR = 2.21, 95% CI: 1.11–4.40; *p* = 0.02).

In terms of infant factors, only one factor was independently associated with the risk of introducing core foods earlier than 17 weeks: mothers who were exclusively formula-feeding their infants at four weeks postpartum had three times the odds of introducing core foods early (adjOR = 3.26, 95% CI: 1.99–5.33; *p* < 0.001) compared to those who were fully breastfeeding at four weeks postpartum. None of the other infant factors, such as infant gender, gestational age, were associated with early introduction of core foods.

### 3.2. Predictors of Early Introduction of Discretionary Foods

In the univariable model, a variety of family and infant factors were associated with early introduction of discretionary foods i.e., before 52 weeks of age (Table 3). Older (OR = 0.94, 95% CI: 0.89–1.00; *p* = 0.05) and university-educated mothers (OR = 0.07, 95% CI: 0.01–0.58; *p* = 0.01) were less likely to introduce discretionary foods early. Women whose partners migrated to Australia from other English-speaking countries were less likely to introduce discretionary foods early (OR = 0.20, 95% CI: 0.08–0.53; *p* = 0.001). Mothers residing in areas considered to be most disadvantaged had the highest odds of introducing discretionary foods early (OR = 3.68, 95% CI: 1.29–10.50; *p* = 0.01). Regarding infant factors, girls were more likely to be introduced discretionary foods early compared to boys (OR = 1.96, 95% CI: 1.01–3.78; *p* = 0.03).

Table 3 presents the adjOR for factors that independently predict early introduction of discretionary foods. Compared to women whose partners were born in Australia, the women whose partners migrated from other English-speaking countries had the lowest odds of introducing discretionary foods to their infants early (adjOR = 0.18, 95% CI: 0.07–0.49; *p* = 0.001). Furthermore, women whose partners migrated from non-English-speaking countries also had lower odds of introducing discretionary foods early (adjOR = 0.42, 95% CI: 0.19–0.94; *p* = 0.03). The odds of introducing discretionary foods before 52 weeks increased as the social gradient declined i.e., mothers residing in areas considered to be most disadvantaged had more than three times the odds of introducing discretionary foods early compared to those living in the least disadvantaged areas (adjOR = 3.72, 95% CI: 1.17–11.78; *p* = 0.02). None of the infant factors, such as infant gender, parity, etc. were associated with early introduction of discretionary foods.

## 4. Discussion

Despite the inconsistencies between the existing guidelines related to the timing of introducing complementary foods, all guidelines agree that complementary foods should not be introduced before 17 weeks of an infant’s age [2,3,37]. Moreover, the type of foods to be introduced is also very important i.e., foods with high fat, salt, and/or sugar content and those with poor nutritional value should be avoided before 12 months of age [14,17]. This study describes the complementary feeding (particularly the age of introduction of core and discretionary foods) practices of socioeconomically disadvantaged and ethnically diverse population residing in SWS, and the family and infant characteristics influencing the early introduction of core and discretionary foods amongst infants.

In the present study, 12% of infants received core foods before 17 weeks of age. The prevalence of early introduction of core foods in the present study is relatively low in comparison to other national and international studies [21,38,39,40,41,42,43]. Mannan et al. [21] reported that almost 82% of mothers based in SWS introduced foods to their infants before four months of age. Another study reported that 33% of Australian infants were receiving foods by four months [38]. Furthermore, this percentage is considerably lower than the estimates from an international cohort study called the Gemini twin birth cohort study conducted in United Kingdom (UK) [16], which reported that 30.5% of infants were introduced to core foods before four months of age.

Despite the existing Australian Infant Feeding Guidelines that recommend that discretionary foods should be avoided before 12 months of age [5,17], in the present study the exposure to discretionary foods started at a very early age (i.e., median age was 28 weeks) and that the majority (95%) of the infants received such foods in the first year of life. Ha et al. [10] in their birth-cohort study involving South Australian infants reported that one in five infants were given sugar-based foods or drinks between six to nine months age. Another Australian cohort study, the PIFS-II, reported that 64% of infants were given discretionary foods by 52 weeks postpartum [44]. Cohort data from a UK-based study [16] showed that 74% of infants were given discretionary foods before 10 months of age. Furthermore, in the present study, the peak age of introduction of both core and discretionary foods was 26 weeks which indicates that most mothers transitioned from breastfeeding or formula-feeding to complementary feeding as per the guidelines i.e., around 6 months or 26 weeks (1); however their food choices did not comply with the recommendations. This finding emphasises the need for effective population-based health promotion and education initiatives to discourage parents from giving unhealthy snacks and sugar-sweetened foods or beverages to their infants since these are the leading cause of common chronic diseases such as diabetes, obesity and ECC.

### 4.1. Predictors of Early Introduction of Core Foods

The present study identified a strong influence of certain family and infant factors on the early introduction of core foods. In accordance with previous research [10,16,40,45,46], the current study found that young and less educated women were more likely to introduce core foods to their infants before 17 weeks of age. Hence, this finding highlights the need to focus on young and less educated mothers to develop appropriate complementary feeding practices related to the timing as well as types of complementary foods to be introduced to their babies.

The employment status of the mother was a strong determinant for early introduction of core foods i.e., mothers who were working (regardless of the type of employment) when their child was 17 weeks old were more likely to introduce core foods early in comparison to non-working mothers. Previous studies did not find any association between maternal employment and early introduction of solid foods specifically [40,45]. For current study analyses, a composite variable was used that took into account the different types of employment i.e., not working, causal, part-time and full-time. Studies using the same composite variable were limited, hence making the comparison difficult. A study by Kuo et al. [47] reported that full-time working mothers had the highest percentage of introducing solid foods early compared to those working part-time or unemployed, but the differences were insignificant. Conversely, in the present study, mothers who were working part-time had the highest odds of introducing core foods early.

Mother’s country of birth was significantly related with the early introduction of core foods. Compared to the mothers born in Australia, those who were born in other English-speaking countries were more likely to introduce core foods to their infants before 17 weeks of age. Mothers who migrated to Australia from non-English speaking countries were less likely to introduce core foods early compared to Australia-born women. Early introduction of solid foods by Australia-born mothers was also reported by other local studies [31,48]. In studies performed in France and Germany, mothers born overseas introduced complementary foods earlier then mothers born locally [45,49]. Griffith et al. [50] reported that white women born in Britain or Ireland were most likely to introduce solid foods before 4-months of infant’s age followed by white women from other European or other countries whereas, mothers born in other countries had lower odds of introducing solid foods earlier. The probable reason for migrant women from non-English speaking countries exhibiting better complementary feeding practices is that they tend to have a better support system since they receive advice from multiple sources such as family members (mothers, mother-in-law, or even maternal/paternal grandmothers) [51] and health professionals of the same cultural background [52].

Mothers who fully formula-fed their babies at four-weeks postpartum were more likely to introduce core foods before 17 weeks of age compared to mothers exclusively breastfeeding. The association between early introduction of complementary foods and formula feeding has been reported in previous studies [53,54]. Exclusive formula feeding is considered to impair the appetite’s self-regulatory mechanism which results in infants asking for solid foods earlier [55].

### 4.2. Predictors of Early Introduction of Discretionary Foods

The majority (95%) of children were given discretionary foods before 52 weeks of age. In terms of predictors, the estimates drawn from Kaplan–Meier (KM) curves showed that there was little important variation in the family and infant-related factors across the study participants who introduced discretionary foods before 52 weeks. Therefore, it is advisable to design and implement population-based approaches and policy-level interventions targeting the high prevalence of early introduction of discretionary foods, since these may be effective across wide population groups.

Based on multivariable analyses, the SES and partner’s country of birth had a significant impact on early introduction of discretionary foods. The present study findings support the concept of social gradient in health [56], although the variation between SES groups was small. Socio-economic status such as low income may impact the health of the family members and it also affects their dietary and lifestyle choices [57]. Children born to mothers living in most disadvantaged areas within the SWS region, were more likely to be introduced discretionary foods early. Ha et al. [10] also reported a significant impact of SES on early introduction of sugar-based foods. The disparity in infant feeding practices in relation to SES can possibly increase health inequalities in later years of life [58].

The country of birth of womens’ partners also had a significant influence on early introduction of discretionary foods to infants. Women whose partners were born in Australia were most likely to introduce discretionary foods before 52 weeks. Comparison with other studies is unfortunately limited, primarily because no other study examined the influence of partners’ country of birth on the infant feeding practices particularly related to timing of discretionary foods introduction. Nevertheless, the partners’ support is a key determinant of infant feeding decisions and practices [24,59]. Walsh et al. [60] in their study involving Australian father-infant dyads reported a strong association between fathers’ and children’s intake of snacks and SSBs. The association observed in the current study demonstrates the prospective significance of including fathers or partners in interventions targeted towards improving infant feeding practices [61].

The present study provides valuable evidence collected through population-based research to determine the level of compliance with the Australian infant feeding recommendations particularly highlighting the poor infant feeding practices related to discretionary foods. This study also demonstrates the social gradients in complementary feeding practices and the importance of a partner’s role in such practices. Research related to infant feeding has shown that dietary recall is to some extent unreliable, and the accuracy of recall diminishes with increasing time gap [62,63]. However, in the present study, the data were collected longitudinally i.e., soon after birth followed by three additional intervals within the first year of life, thereby minimising the chances of heaping of data and recall bias [64]. Timing of introduction was measured in weeks, which allows for precise measurement of the time of event (i.e., age of introducing core and discretionary foods respectively).

The present study has few limitations. The timings of introduction of core and discretionary foods were measured, however, the amount consumed was not measured. The key food/drink items within the core and discretionary food groups were included in the diet diary, however, there is a possibility that some food/drink items stated in the Australian Dietary Guidelines might have been omitted. The study sample consisted of women who underwent delivery in public hospitals located in SWS and, therefore, might not be considered to be representative of either SWS or NSW or the Australian population as a whole. The study outcome was self-reported similar to other studies on this topic and, therefore, there might be chances of social desirability bias even though the recall period was short.

## 5. Conclusions

The present study provides an insight into the infant feeding practices of mothers residing in the South West Sydney region which is one of Australia’s most ethnically diverse areas with high levels of social disadvantage. The study results suggest a better compliance with infant feeding guidelines in relation to the age of introduction of core foods. However, a very high number of infants were given discretionary foods in their first year of life. Mothers of young age, those with lower levels of education, those who are working while the child is very young, and those who fully formula-feed their babies at four weeks postpartum are more likely to introduce core foods early. These population groups should be targeted to promote appropriate complementary feeding practices. Furthermore, mothers living in the most disadvantaged neighbourhoods had the highest likelihood of introducing discretionary foods early and, therefore, should be educated to make informed decisions regarding the dietary choices for their infants.

## Figures and Tables

**Table 1:** Family and infant characteristics associated with early introduction of core and discretionary foods.

Characteristics	Core Foods (*n* = 934) ^a^		Discretionary Foods (*n* = 900) ^b^	
	<17 weeks	≥17 weeks	<52 weeks	≥52 weeks
Family Characteristics *	**N (%)**	**N (%)**	**N (%)**	**N (%)**
Maternal education				
Left school before 12	30 (17.9%)	138 (82.1%)	157 (99.4%)	1 (0.6%)
Completed school	34 (17.7%)	158 (82.3%)	182 (97.9%)	4 (2.1%)
College/TAFE	20 (11.8%)	150 (88.2%)	157 (95.1%)	8 (4.9%)
University	28 (6.9%)	376 (93.1%)	362 (92.6%)	29 (7.4%)
Maternal marital status				
Married	72 (9.8%)	662 (90.2%)	672 (94.8%)	37 (5.2%)
Living with partner	18 (16.4%)	92 (83.6%)	101 (96.2%)	4 (3.8%)
Single	22 (24.4%)	68 (75.6%)	85 (98.8%)	1 (1.2%)
Maternal work status at 17-weeks				
Not working	93 (11.3%)	733 (88.7%)	-	-
Casual	5 (20.0%)	20 (80.0%)	-	-
Part-time	10 (22.7%)	34 (77.8%)	-	-
Full-time	4 (10.8%)	33 (89.2%)	-	-
Maternal work status at 52-weeks				
Not working	-	-	461 (95.6%)	21 (4.4%)
Casual	-	-	33 (97.1%)	1 (2.9%)
Part-time	-	-	209 (94.6%)	12 (5.4%)
Full-time	-	-	111 (94.1%)	7 (5.9%)
Maternal country of birth				
Australia born	65 (14.9%)	372 (85.1%)	410 (96.5%)	15 (3.5%)
Other English-speaking countries	16 (26.7%)	44 (73.3%)	52 (89.7%)	6 (10.3%)
Non-English-speaking countries	31 (7.1%)	406 (92.9%)	396 (95.0%)	21 (5.0%)
Partner country of birth				
Australia born	52 (14.0%)	319 (86.0%)	349 (96.7%)	12 (3.3%)
Other English-speaking countries	9 (15.5%)	49 (84.5%)	48 (85.7%)	8 (14.3%)
Non-English-speaking countries	33 (7.8%)	389 (92.2%)	389 (95.3%)	19 (4.7%)
Index for relative socioeconomic advantage and disadvantage				
Least disadvantaged	16 (7.2%)	205 (92.8%)	201 (93.9%)	13 (6.1%)
Low disadvantaged	24 (15.0%)	136 (85.0%)	141 (90.4%)	15 (9.6%)
Moderately disadvantaged	7 (23.3%)	23 (76.7%)	27 (90.0%)	3 (10.0%)
Highly disadvantaged	27 (12.3%)	193 (87.7%)	204 (97.1%)	6 (2.9%)
Most disadvantaged	38 (12.5%)	265 (87.5%)	285 (98.3%)	5 (1.7%)
Maternal smoking during pregnancy				
No	95 (10.8%)	785 (89.2%)	812 (95.5%)	38 (4.5%)
Yes	16 (30.2%)	37 (69.8%)	45 (91.8%)	4 (8.2%)
Maternal alcohol consumption during pregnancy				
No	95 (11.4%)	737 (88.6%)	762 (95.0%)	40 (5.0%)
Yes	16 (16.0%)	84 (84.0%)	94 (97.9%)	2 (2.1%)
Parity				
Primiparous	57 (12.3%)	408 (87.7%)	421 (94.2%)	26 (5.8%)
Multiparous	55 (11.7%)	414 (88.3%)	437 (96.5%)	16 (3.5%)
**Infant characteristics**				
Infant gender				
Male	59 (12.4%)	418 (87.6%)	433 (93.9%)	28 (6.1%)
Female	53 (11.6%)	404 (88.4%)	425 (96.8%)	14 (3.2%)
Infant gestational age				
Pre-term	5 (7.1%)	65 (92.9%)	64 (97.0%)	2 (3.0%)
Full-term	107 (12.4%)	757 (87.6%)	794 (95.2%)	40 (4.8%)
Infant birth-weight				
≥2500g	106 (12.0%)	781 (88.0%)	816 (95.3%)	40 (4.7%)
<2500g	6 (12.8%)	41 (87.2%)	42 (95.45%)	2 (4.55%)
Method of delivery				
Vaginal	79 (12.1%)	573 (87.9%)	595 (95.0%)	31 (5.0%)
Caesarean	32 (11.4%)	249 (88.6%)	262 (96.0%)	11 (4.0%)
Infant feeding method at 4-weeks age				
Only breastfeeding	52 (9.0%)	523 (91.0%)	526 (95.0%)	28 (5.0%)
Only formula feeding	43 (28.3%)	109 (71.7%)	140 (96.6%)	5 (3.4%)
Both breastfeeding and formula feeding	16 (7.8%)	189 (92.2%)	190 (95.5%)	9 (4.5%)

^a,b^ The total of the categories might not always add up to 934 and 900 due to missing or incomplete data for some item * Maternal age is a continuous variable therefore; the percentages have not been reported. N: sample size. TAFE: Technical and Further Education.

**Table 2:** Unadjusted and adjusted odds ratio (OR) for early introduction of core foods (<17 weeks).

Characteristics	Unadjusted Odds Ratio ^a^			Adjusted Odds Ratio ^b^		
	OR (95% CI)	*p*-Value	Overall *p*-Value	OR (95% CI)	*p*-Value	Overall *p*-Value
Family Characteristics **						
Maternal Age	0.90 (0.87–0.94)	<0.001	<0.001	0.92 (0.88–0.96)	<0.001	<0.001
Maternal education						
Left school before 12	1.00					
Completed school	0.98 (0.57–1.70)	0.990		1.24 (0.68–2.25)	0.472	
College/TAFE	0.61 (0.33–1.13)	0.117	<0.001	0.75 (0.38–1.47)	0.414	0.037
University	0.34 (0.19–0.59)	<0.001		0.53 (0.28–0.99)	0.047	
Maternal marital status						
Married	1.00			Not retained in the final model		
Living with partner	1.79 (1.02–3.15)	0.040	<0.001			
Single	2.97 (1.73–5.09)	<0.001				
Maternal work status at 17-weeks						
Not working	1.00					
Casual	1.97 (0.72–5.37)	0.185		2.0 (0.67–5.99)	0.212	
Part-time	2.31 (1.10–4.84)	0.025	0.126	3.42 (1.54–7.62)	0.003	0.017
Full-time	0.95 (0.33–2.75)	0.933		1.25 (0.41–3.80)	0.688	
Maternal country of birth						
Australia born	1.00					
Other English-speaking countries	0.31 (0.11–0.85)	0.023	0.099	2.21 (1.11–4.40)	0.023	<0.001
Non-English-speaking countries	0.68 (0.35–1.35)	0.282		0.53 (0.32–0.86)	0.011	
Partner country of birth						
Australia born	1.00			Not retained in the final model		
Other English-speaking countries	0.20 (0.08–0.53)	0.001	0.010			
Non-English-speaking countries	0.70 (0.33–1.47)	0.351				
Index for relative socioeconomic advantage and disadvantage						
Least disadvantaged	1.00			Not retained in the final model		
Low disadvantaged	2.26 (1.15–4.41)	0.017				
Moderately disadvantaged	3.89 (1.45–10.46)	0.007	0.044			
Highly disadvantaged	1.79 (0.93–3.42)	0.078				
Most disadvantaged	1.83 (0.99–3.38)	0.051				
Maternal smoking during pregnancy						
No	1.00					
Yes	3.57 (0.95–5.70)	<0.001	<0.001	Not retained in the final model		
Maternal alcohol consumption during pregnancy						
No	1.00			Not retained in the final model		
Yes	1.47 (0.83–2.62)	0.184	0.198			
Infant Characteristics **						
Infant gestational age						
Pre-term	1.00			Not retained in the final model		
Full-term	1.83 (0.72–4.66)	0.201	0.166			
Infant feeding method at 4 weeks of age						
Only breastfeeding	1.00					
Only formula feeding	3.96 (2.52–6.24)	<0.001	<0.001	3.26 (1.99–5.33)	<0.001	<0.001
Both breastfeeding and formula feeding	0.85 (0.47–1.52)	0.59		0.91 (0.49–1.69)	0.787	

Note: Backward stepwise model with dichotomous outcome of (0: <17-weeks; 1: ≥17-weeks). ^a^ All the variables in the Unadjusted model were variables which had a *p*-value of <0.25. ** child gender, birth-weight, method of delivery, and parity had a *p*-value >0.25; therefore, were not included in the unadjusted model. ^b^ Adjusted for maternal marital status, partners’ country of birth, index of relative socio-economic advantaged and disadvantage, mother’s smoking during pregnancy, mother’s alcohol consumption during pregnancy and infant gestational age. OR: odds ratio, 95% CI: 95% confidence interval.

**Table 3:** Unadjusted and adjusted odds ratio for early introduction of discretionary foods (<52-weeks).

Characteristics	Unadjusted Odds Ratio ^a^			Adjusted Odds Ratio ^b^		
	OR (95% CI)	*p*-Value	Overall *p*-Value	OR (95% CI)	*p*-Value	Overall *p*-Value
Family Characteristics **						
Maternal Age	0.94 (0.89–1.00)	0.055	0.055	Not retained in the final model		
Maternal education						
Left school before 12	1.00			Not retained in the final model		
Completed school	0.28 (0.03–2.61)	0.270				
College/TAFE	0.12 (0.01–1.01)	0.051	<0.001			
University	0.07 (0.01–0.58)	0.013				
Maternal marital status						
Married	1.00			Not retained in the final model		
Living with partner	1.39 (0.48–3.98)	0.54	0.134			
Single	4.68 (0.63–34.54)	0.13				
Maternal country of birth						
Australia born	1.00					
Other English-speaking countries	0.31 (0.11–0.85)	0.023	0.098	Not retained in the final model		
Non-English-speaking countries	0.68 (0.35–1.35)	0.282				
Partner country of birth						
Australia born	1.00					
Other English-speaking countries	0.20 (0.08–0.53)	0.001	0.009	0.18 (0.07–0.49)	0.001	0.002
Non-English-speaking countries	0.70 (0.33–1.47)	0.351		0.42 (0.19–0.94)	0.035	
Index for relative socioeconomic advantage and disadvantage						
Least disadvantaged	1.00					
Low disadvantaged	0.60 (0.28–1.31)	0.207		0.60 (0.25–1.43)	0.254	
Moderately disadvantaged	0.58 (0.15–1.55)	0.421	0.001	0.39 (0.09–1.55)	0.183	0.004
Highly disadvantaged	2.19 (0.81–5.89)	0.118		2.06 (0.71–5.92)	0.179	
Most disadvantaged	3.68 (1.29–10.50)	0.015		3.72 (1.17–11.78)	0.025	
Maternal alcohol consumption during pregnancy						
No	1.00			Not retained in the final model		
Yes	2.46 (0.58–10.3)	0.218	0.159			
Parity						
Primiparous	1.00			Not retained in the final model		
Multiparous	1.68 (0.89–3.18)	0.018	0.102			
Infant Characteristics **						
Infant gender						
Male	1.00			Not retained in the final model		
Female	1.96 (1.01–3.78)	0.044	0.038			

Note: Backward stepwise model with dichotomous outcome of (0: <52-weeks; 1: ≥52-weeks). ^a^ All the variables in the unadjusted model were variables which had a *p*-value of <0.25. ** maternal work status at 52 weeks, infant gestational age, birth-weight, method of delivery, mother’s smoking during pregnancy, infant feeding method at 4 weeks of age, had a *p*-value >0.25; therefore, were not included in the unadjusted model. ^b^ Adjusted for maternal age, education, marital status, country of birth, partners’ country of birth, alcohol consumption status of mother during pregnancy, infant gender and parity. OR: odds ratio, 95% CI: 95% confidence interval.
